# Value of an Intraoral Camera in Evaluating Restorations and Plaque in a Simulated Environment: Observational Experiences of Dentists in Pediatric Contexts to Establish a Proof of Concept

**DOI:** 10.7759/cureus.72012

**Published:** 2024-10-21

**Authors:** Christine Schiller, Huong Ho, Takanori Sobue, Bina Katechia, Aditya Tadinada

**Affiliations:** 1 Oral and Maxillofacial Radiology, University of Connecticut School of Dental Medicine, Farmington, USA; 2 Oral and Maxillofacial Radiology, University of Connecticut Health, Farmington, USA; 3 Periodontology, University of Connecticut Health, Farmington, USA; 4 Pediatric Dentistry, University of Connecticut Health, Farmington, USA

**Keywords:** caries, diagnosis, intraoral camera, margins, plaque, restorations

## Abstract

Access to the posterior maxillary and mandibular teeth is a significant challenge while evaluating plaque, gingiva, teeth, restorations, and crown margins. It is tougher when there are also limitations of mouth opening, small mouths, or when patients are not cooperative. The evolution of small intraoral cameras appears to be a promising solution - intraoral cameras fit into the mouth and deliver live, high-resolution video feeds of the intraoral areas. There are not many well-designed studies that have tested the value addition of an intraoral camera in pediatric patient scenarios. The objective of this study is to evaluate the value addition of an intraoral camera compared to conventional evaluation using a mouth mirror and explorer in its ability to evaluate the posterior maxillary and mandibular teeth to study common pediatric dental scenarios, namely, evaluation of plaque, margins of restorations, margins of a stainless-steel crown, and permanent tooth eruption. Pediatric typodonts were utilized to simulate a representation of common intraoral scenarios in the pediatric patient. Reviewers first examined typodonts with a mouth mirror and explorer and then with an intraoral camera to determine the visibility and clarity of the simulated scenarios. This process was then repeated two weeks later for evaluation of intra-rater reliability. For all the parameters evaluated using the conventional method, all four evaluators marked “not visible” as the most chosen option. For examination with the intraoral camera, all evaluators marked “clearly visible” as the most chosen option. All the raters agreed that the intraoral camera added a significant advantage in clinical examinations, especially in hard-to-visualize areas. The results also show a high intra-rater reliability. The results of this proof-of-concept study show that the intraoral camera adds significant value to the visualization of posterior maxillary and mandibular arches for evaluation of cavity preparations, restorations, tooth eruption, and identifying plaque in pediatric typodonts.

## Introduction

Intraoral cameras are increasingly becoming significant in dental practices for their ability to enhance a clinician’s visibility in the patient’s mouth, especially in the hard-to-visualize posterior maxillary and mandibular teeth. Pentapati et al. stated that these cameras, first established in 1987, have significantly evolved in size, function, and capabilities and provide numerous benefits to dental professionals [1]. The hard-to-visualize areas in the mouth are typically the posterior teeth and better visualization of these areas leads to better diagnoses. Intraoral cameras allow for a quick swipe of the areas of interest and provide a live video feed of the areas being swiped within a short acquisition time. This can help in the detection of caries and gingivitis with higher accuracy than standard clinical examinations, as well as make the patients a part of the treatment plan, leading to patient compliance, motivation, and education. These images can be archived for patient records and insurance claims, making them a valuable asset in dental practices [1-5]. From a pediatric perspective, several studies have evaluated intraoral camera screenings in school settings, discussed patient education on topics such as oral hygiene, and used a Likert scale to rate overall camera experience [1-4]. Current literature does not have many well-designed studies that have evaluated the value of an intraoral camera in typical clinical scenarios in pediatric populations, where patient compliance may be decreased and therefore limit the potential for comprehensive evaluation. In this proof-of-concept study using pediatric typodonts, we simulated typical pediatric dental scenarios and aimed to assess whether the utilization of an intraoral camera has any significant advantage over standard clinical oral examination in a variety of pediatric treatment scenarios. Perspectives of dental clinicians were assessed using a Likert scale comparing typical oral examination with the intraoral camera. We hypothesized that, upon comparing standard clinical examination to examination with an intraoral camera, clinicians would rate the intraoral camera higher in terms of accessibility and visibility of posterior maxillary and mandibular teeth in typodont-simulated pediatric patient scenarios.

## Technical report

Materials and methods

**Figure 1:**
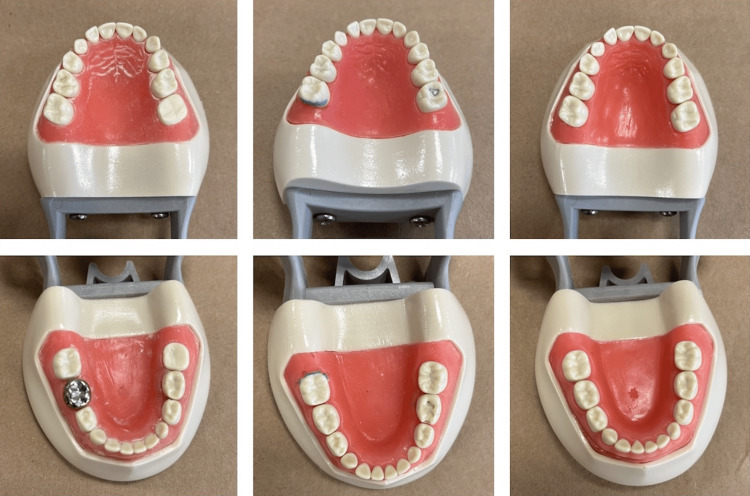
Pediatric typodonts with maxillary and mandibular simulated scenarios

**Figure 2:**
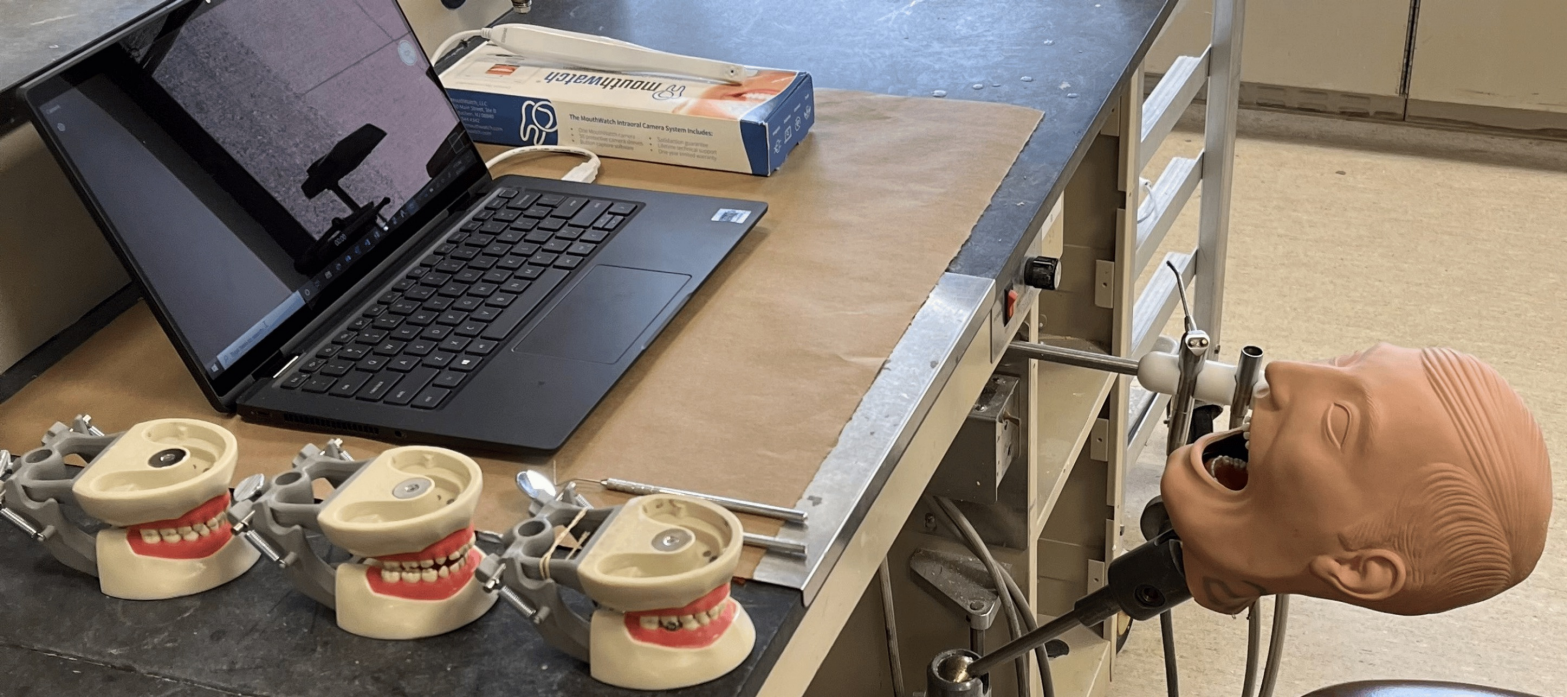
Experimental set-up with the intraoral camera and typodonts

This study utilized pediatric typodonts to simulate dental scenarios in posterior maxillary and mandibular teeth in each quadrant (Figure 1). Scenarios prepared included plaque simulation with disclosing solution, class-2 cavity preparation, amalgam and composite restorations, stainless-steel crown, and tooth eruption simulation. The typodonts were mounted onto a pole mannequin and simulated a real-life scenario relative to mouth opening and access (Figure 2). Four board-certified dentists with no previous experience using an intraoral camera were asked to evaluate the scenarios both by visual examination (explorer and mirror) and with the help of the intraoral camera using a three-point modified Likert scale, with the completion of evaluations twice with an interval of at least two weeks between each session. The three-point Likert scale included both mandibular and maxillary scenarios, as well as categories for buccal, lingual/palatal, interproximal - mesial, and interproximal - distal surfaces with the following rating options: (1) clearly visible; (2) visible, not clear; and (3) not visible. These ratings were asked first regarding visual examination by mirror and explorer and subsequently for utilization of the intraoral camera. A general question was also asked about whether the utilization of an intraoral camera as opposed to a mouth mirror provided a significant advantage in clinical examination, with the Likert options (1) agree, (2) neutral, and (3) disagree. Statistical analysis included an intra-rater agreement for each of the simulated clinical scenarios.

Results

**Figure 3:**
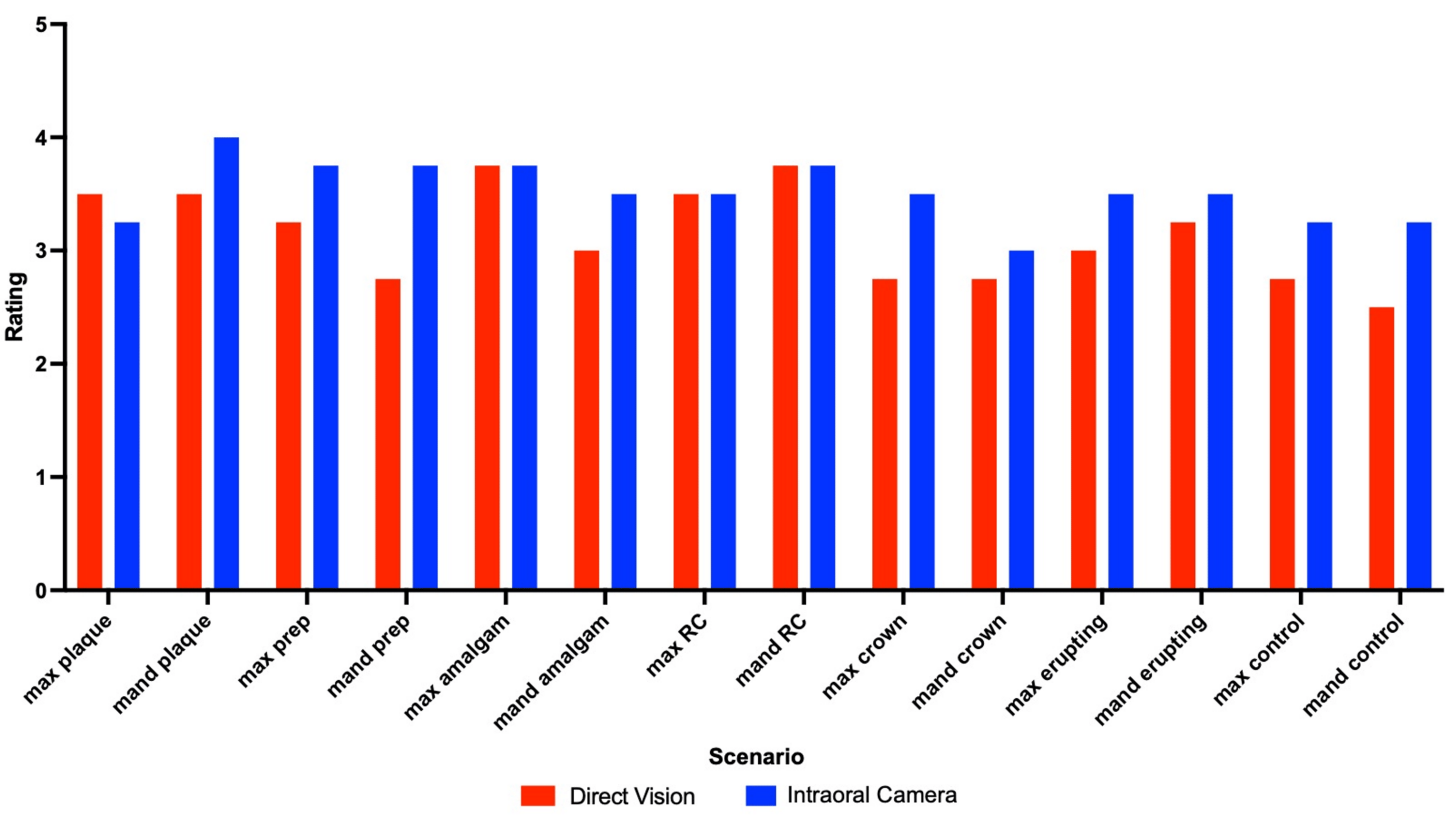
Average ratings across all raters per typodont scenario comparing direct vision with the intraoral camera

Upon comparing the visualization of maxillary posterior teeth using either traditional clinical examination or an intraoral camera, all raters concluded that examination with the intraoral camera, on average, provided better visualization. Scenarios were analyzed by determining how many out of four tooth surfaces were "clearly visible" with either mouth mirror and explorer or intraoral camera. Among all scenarios and all raters, an average of 3.14 out of 4 tooth surfaces were rated as "clearly visible" utilizing a mouth mirror and explorer versus 3.52 out of 4 tooth surfaces rated as "clearly visible" utilizing the intraoral camera (Figure 3). Intra-rater reliability was performed utilizing Cohen’s kappa analysis and found that there was fair to substantial agreement among raters between their first and second ratings with an interval of two weeks in between ratings, with two raters having moderate agreement (0.427 and 0.405), one with fair agreement (0.248), and one with substantial agreement between ratings (0.664) (Table 1). These results display the benefit of utilizing an intraoral camera in pediatric examinations due to the higher average number of tooth surfaces rated as "clearly visible" among all raters when using the camera as opposed to a typical examination with a mirror and explorer. In addition to this conclusion, intra-rater reliability reveals that the results were consistent among raters' first and second evaluations.

**Table 1:** Intra-rater reliability

Intra-rater reliability			
Rater 1	Rater 2	Rater 3	Rater 4
0.664	0.427	0.405	0.248

## Discussion

The results of this proof-of-concept study show that an intraoral camera had a significant impact on the ability of dentists to clearly visualize some common pediatric scenarios within posterior teeth. Upon dividing teeth into four surfaces and determining how many of those surfaces were considered to be "clearly visible" (as opposed to visible but not clear or not visible at all), there was a fairly noticeable difference between the scores with and without the utilization of the intraoral camera. When examining the simulated patient mouth with the intraoral camera, an average of 3.52 out of 4 tooth surfaces were determined to be "clearly visible" as compared to 3.14 out of 4 tooth surfaces with a prototypical mouth mirror and explorer. These averages were accumulated from the ratings of four board-certified dentists. In addition, when the dentists were asked at least two weeks later to rate the same scenarios a second time, their second ratings had high agreement with the first. All raters showed fair to substantial agreement utilizing Cohen’s kappa analysis.

The results of our study can provide insight into the future of clinical examination, especially in pediatric practice. Pediatric patients bring additional challenges to intraoral examination due to small mouths, lower compliance, and lack of education on proper oral hygiene. The intraoral camera can be a valuable tool in the practitioner’s office because of its ability to visualize hard-to-reach posterior areas of the mouth lacking adequate light source availability. The camera is small and lightweight, so it can fit into the small corners and crevices of the adolescent mouth, and it contains a light to illuminate the field. In addition, decreased compliance by pediatric patients can be an issue for examination with a mirror and explorer, which may take longer to complete thoroughly. The intraoral camera allows one to take pictures, as well as videos, which can be taken quickly intraorally and then subsequently analyzed out of the patient’s mouth. Videos that may be taken of areas of questionable caries, defective restorations, or any intraoral inflammation can be played on a loop for the dentist to watch without having to constantly enter the child’s mouth. The ability to look at these media types outside of the mouth can account for the decreased compliance and patience that is encountered in pediatric practices. Furthermore, the intraoral camera can be used as an educational tool for patients and their parents. The camera can be mirrored onto any laptop or other screen for the patient and/or parent to watch as the dentist shows an increased presence of plaque in certain areas of the mouth, inflammation, or a demonstration of proper oral hygiene such as brushing or flossing. The intraoral camera has many possible uses, which can benefit both the patient and the provider.

Taking into account previous literature on this topic, it can be agreed upon that continued research into the benefit of intraoral cameras is recommended. A systematic review conducted by Pentapati et al. found that intraoral cameras provide a wide range of benefits including diagnosis of clinical conditions, as well as significant education to all patient populations, including those that wear appliances such as dentures or others learning about unhealthy gum tissue and peri-implantitis [1]. Forgie et al. examined the ability of an intraoral camera to diagnose occlusal caries in the premolar and molar teeth gathered from dentists throughout Scotland; the utilization of natural teeth revealed the camera's ability to analyze natural tooth structure, unlike our typodont models. However, it did not examine pediatric scenarios in particular, which our study aimed to do [3]. As an example within real patient populations, Murrell et al. evaluated student, doctor, and patient opinions on the benefit of an intraoral camera as an aid when completing posterior crowns. The study determined that there was generally a positive outlook on the intraoral camera but, again, does not highlight the particular benefit of the camera in pediatric examinations [4]. Our study aimed to build upon this literature with a specific stance on the positive aspects of using an intraoral camera in pediatric populations.

Since our results are from a proof-of-concept study utilizing mannequin heads and pediatric typodonts, we are unable to make any conclusions about the use of the intraoral camera in real patients nor are we able to extrapolate our data to include a real pediatric patient population. We do, however, have promising results, which provide an appropriate and substantial rationale for continuing our research on human populations. Therefore, future studies would include real pediatric patients with a variety of intraoral scenarios, including the ones we utilized in this study. We would also prefer to go forward with these studies using very large groups of data, as well as increased numbers of raters so that we have high reliability in our studies and eliminate any potential observer bias.

## Conclusions

In this proof-of-concept study, the intraoral camera provided a significant advantage to dentists for clinical examination in both maxillary and mandibular posterior areas, especially geared at pediatric scenarios, such as erupting teeth, cavity preparations, restorations with amalgam and resin composite materials, stainless steel crowns, and plaque-disclosing solution.

This proof-of-concept study could lead the road forward to a variety of applications in dentistry. The intraoral camera is particularly useful in hard-to-access areas and in pediatric age groups where the patients are not cooperative. This tool will be very important in making the patient a valuable stakeholder in the evaluation, diagnosis, and treatment planning stages, leading to a more confident execution of the treatment plan. The pictures and videos recorded by the probe can also serve as records for future reference. Overall, this is a very valuable addition to the dental armamentarium, and more clinical studies can further fortify this concept.
